# The Dual Impact of HIV-1 Infection and Aging on Naïve CD4^+^ T-Cells: Additive and Distinct Patterns of Impairment

**DOI:** 10.1371/journal.pone.0016459

**Published:** 2011-01-26

**Authors:** Tammy M. Rickabaugh, Ryan D. Kilpatrick, Lance E. Hultin, Patricia M. Hultin, Mary Ann Hausner, Catherine A. Sugar, Keri N. Althoff, Joseph B. Margolick, Charles R. Rinaldo, Roger Detels, John Phair, Rita B. Effros, Beth D. Jamieson

**Affiliations:** 1 Department of Medicine, UCLA AIDS Institute, David Geffen School of Medicine, University of California Los Angeles, Los Angeles, California, United States of America; 2 Department of Pathology and Laboratory Medicine, UCLA AIDS Institute, University of California Los Angeles, Los Angeles, California, United States of America; 3 Department of Biostatistics, School of Public Health, University of California Los Angeles, Los Angeles, California, United States of America; 4 Department of Epidemiology, Johns Hopkins Bloomberg School of Public Health, Baltimore, Maryland, United States of America; 5 Department of Molecular Microbiology and Immunology, Johns Hopkins Bloomberg School of Public Health, Baltimore, Maryland, United States of America; 6 Graduate School of Public Health, University of Pittsburgh, Pittsburgh, Pennsylvania, United States of America; 7 Department of Epidemiology, School of Public Health, University of California Los Angeles, Los Angeles, California, United States of America; 8 Feinberg School of Medicine, Northwestern University, Chicago, Illinois, United States of America; New York University, United States of America

## Abstract

HIV-1-infected adults over the age of 50 years progress to AIDS more rapidly than adults in their twenties or thirties. In addition, HIV-1-infected individuals receiving antiretroviral therapy (ART) present with clinical diseases, such as various cancers and liver disease, more commonly seen in older uninfected adults. These observations suggest that HIV-1 infection in older persons can have detrimental immunological effects that are not completely reversed by ART. As naïve T-cells are critically important in responses to neoantigens, we first analyzed two subsets (CD45RA^+^CD31^+^ and CD45RA^+^CD31^-^) within the naïve CD4^+^ T-cell compartment in young (20–32 years old) and older (39–58 years old), ART-naïve, HIV-1 seropositive individuals within 1–3 years of infection and in age-matched seronegative controls. HIV-1 infection in the young cohort was associated with lower absolute numbers of, and shorter telomere lengths within, both CD45RA^+^CD31^+^CD4^+^ and CD45RA^+^CD31^-^CD4^+^ T-cell subsets in comparison to age-matched seronegative controls, changes that resembled seronegative individuals who were decades older. Longitudinal analysis provided evidence of thymic emigration and reconstitution of CD45RA^+^CD31^+^CD4^+^ T-cells two years post-ART, but minimal reconstitution of the CD45RA^+^CD31**^-^**CD4^+^ subset, which could impair *de novo* immune responses. For both ART-naïve and ART-treated HIV-1-infected adults, a renewable pool of thymic emigrants is necessary to maintain CD4^+^ T-cell homeostasis. Overall, these results offer a partial explanation both for the faster disease progression of older adults and the observation that viral responders to ART present with clinical diseases associated with older adults.

## Introduction

The lifespan of an HIV-1-infected North American or European individual is shortened by an average of 10 years, despite antiretroviral therapy (ART) [1]. Many of the causes of morbidity and mortality in these individuals are similar to those more commonly observed in uninfected older adults (50–65 years of age) and the elderly (>65 years of age), and include frailty [2], non**-**Hodgkin's lymphoma [3], anal and cervical carcinomas [4], [5], osteoporosis [6], [7], liver [8]–[10] and renal impairment [11], cardiovascular disease [12], [13], diabetes [14] and hypertension [14], [15]. The diminished lifespan and higher prevalence of these diseases in HIV-1-infected individuals, in comparison to age-matched uninfected controls, has led to the theory that HIV-1 infection causes accelerated aging in multiple organ systems. As it is not clear whether HIV-1 contributes to age-inappropriate clinical manifestations through mechanisms distinct from aging, a better understanding of the effects of HIV-1 infection and aging on various organ systems is essential to future treatment of HIV-1-infected individuals.

Survival time for HIV-1-infected adults both pre-and post-ART is closely correlated with CD4^+^ T-cell counts. The life-expectancy of an untreated HIV-1-infected individual with 200 CD4^+^ T-cells/mm^3^ is approximately one to two years [16]. An ART-treated, 20-year-old adult with a CD4^+^ T-cell count under 200 cells/mm^3^ at ART initiation is predicted to survive 32 years, compared to 50 years for an age-matched individual who initiates ART at a higher CD4^+^ T-cell count [16]. An increased risk for frailty is also associated with decreased CD4^+^ T-cell counts pre- and post-ART initiation, as is the risk for non-Hodgkin lymphoma [2], [3]. Poor CD4^+^ T-cell recovery upon initiation of ART is also correlated with an increased risk for both AIDS and non-AIDS diseases [17], emphasizing the important role of the CD4^+^ T-cell compartment in maintaining good health.

Although HIV-1 infection of naïve CD4^+^ T-cells occurs at low frequency in comparison to that of activated effector/memory CD4^+^ T-cells, HIV-1 infection is associated with quantitative and qualitative changes within the naïve CD4^+^ T-cell compartment in both children and adults [18]–[21]. In HIV-1-infected adults, a loss of naïve CD4^+^ T-cells precedes the loss of T-cell homeostasis and progression to AIDS [20], and inverted naïve to effector/memory ratios are not always restored upon administration of ART [17], [22]. Since reconstitution of the naïve T-cell compartment contributes to reconstitution of overall CD4^+^ T-cell counts, a continued deficit in naïve CD4^+^ T-cell numbers would have downstream implications for the effector/memory compartment. In addition, functional defects, such as diminished antigen-specific proliferative responses [23], persist in the naïve CD4^+^ T-cell compartment, despite treatment. As naïve CD4^+^ T-cell proliferative responses post-ART predict immune responses to immunization with neoantigens [24], it is possible that an impaired naïve CD4^+^ T-cell compartment may contribute to the clinical observations regarding poor health and age-associated pathologies post-ART.

Aging, in the absence of HIV infection, is also associated with quantitative and qualitative changes within the naïve CD4^+^ T-cell compartment [25]–[27]. Decreased numbers of recent thymic emigrants (RTE), shortened telomeres, hyporesponsiveness to stimulation, decreased proliferative capacity, reduced IL-2 production, alterations in signal transduction and changes in cell surface phenotype [26]–[28] have all been reported. These changes likely contribute to the poor response to vaccines and increased susceptibility to infectious diseases and neoplasms reported for older adults [29]–[31] and possibly also contribute to the more rapid disease progression in HIV-1-infected individuals over 50 years of age.

Many factors, including co-infections and toxicity, or negative side effects, of antiretroviral drugs, are likely to contribute to the decreased lifespan and increased morbidities seen in HIV-1-infected individuals. In the current study, we address the question of whether HIV-1 infection accelerates the aging process by characterizing the effects of HIV-1 infection and aging on the naïve CD4^+^ T-cell compartment. We investigate whether the negative effects of HIV-1 infection and aging on the naïve CD4^+^ T-cell compartment are additive or interactive. To this end, we subdivided the naïve CD4^+^ T-cell compartment into two biologically disparate subsets based on the surface expression of PECAM-1 (CD31), which distinguishes TREC high (CD31^+^) naïve CD4^+^ T-cells from their proliferative progeny, the TREC low (CD31^-^) naïve CD4^+^ T-cell subset [27], [32]. We had previously shown that aging is associated with changes in the relative proportions of these two subsets and with telomere shortening within cells from both subsets [27]. Therefore, in the current study we compared the two naïve CD4^+^ T-cell subsets within younger (20–32 years old) and older (39–58 years old) HIV-1 seropositive (SP) ART-naïve adults relatively early in infection, as well as with HIV-1 seronegative (SN) age-matched adults. Our results suggest that the effects of HIV-1 infection and aging on the the naïve CD4^+^ T-cell compartment are both additive and distinct, and that HIV-1 induced impairments are not fully restored to an age-appropriate status by ART.

## Materials and Methods

### Ethics Statement

All study participants from the cross-sectional study were recruited from the Los Angeles area. This study was approved by the University of California, Los Angeles Medical Institutional Review Board and each participant provided written, informed consent per the approved protocol.

### Participants

Cross-sectional study. Twenty-eight HIV-1 SN participants aged 19–30 years, nineteen SN participants aged 47–60 years, nine HIV-1 SP participants aged 20–32 years, and ten SP participants aged 39–58 years. All SP participants were within 1–3 years of infection by self-report and were treatment-naïve.

Longitudinal study. From the Multicenter AIDS Cohort Study (MACS), a study of the natural and treated history of HIV-1 infection in men who have sex with men [33], [34], we selected ten SP men who initiated ART while enrolled in the MACS. ART is self-reported during the semi-annual MACS study visits. Selection criteria included the following characteristics at the visit prior to initiating ART: an age of 40–50 years, an absolute T-cell count >250 cells/mm^3^ of blood, a viral load >50 copies/ml (90% of those selected had >5000 copies/ml), and a successful response to treatment, defined by a viral load of <400 RNA copies/ml approximately one year post-ART (90% of those selected had a viral load of <50 RNA copies/ml). The average increase in absolute CD4^+^ T-cell count one year post-ART was 147 cells/mm^3^. Ten SN MACS participants, age-matched (within 6 months to a year of age) to the pre-ART donor visit, were selected as controls. We analyzed cryopreserved PBMC provided by these men at 6 months to one year prior to ART initiation and at 1 and 2 years after that time.

### Immunophenotyping and Cell Sorting

For the cross-sectional study, blood was collected into ethylenediaminetetraacetic acid (EDTA)-treated vacutainer tubes (Becton Dickinson, Rutherford, NJ). Whole blood was stained with the following monoclonal antibodies: CD45RA fluorescein isothiocyanate (FITC), CD4 peridinin chlorophyll protein (PerCP^TM^), CD31 phycoerytherin (PE) and CD28 and/or CD27 allophycocyanin (APC), (BD Biosciences Immunocytometry Systems (BDIS), San Jose, CA). Whole blood was stained, lysed, and analyzed as described [35]. PBMC for longitudinal analyses were originally collected in heparin and cryopreserved in liquid nitrogen. After thawing, the PBMC were stained as described above and the cells were washed twice in 1 ml of 1X PBS containing 2% newborn calf serum (NCS) and 0.1% sodium azide. To aid in establishing a viable lymphocyte gate for cryopreserved PBMC, an additional tube stained with 7-AAD, a marker for cellular viability [36], was utilized. Samples were analyzed using a FACSCalibur^TM^ flow cytometer (BDIS) equipped with a 15 mW air-cooled 488 nm argon-ion laser and a red diode laser emitting at ∼635 nm. For each sample, a minimum of 5,000 CD4^+^ lymphocytes were collected. Analysis of the data was performed with CELLQuest^TM^ software (BDIS).

Cell sorting was performed on a FACSAria flow cytometer (BDIS). A singlet gate of side scatter height versus side scatter width was used in addition to the standard forward scatter versus side scatter gating, in order to exclude cell “doublets”. PBMC, both freshly isolated as described [27], and cryopreserved, were stained with CD45RA-FITC, CD31-PE, CD4-APC and CD8 APC-CY7 for cell sorting. The sorts for CD45RA^+^CD31^+bright^CD4^+^ yielded cells that expressed 2-3 times more CD31 than the sorts for CD45RA^+^CD31^+dim^ CD4^+^. The sort gate for CD45RA^+^CD31^+dim^CD4^+^ T-cells was very narrow in order to exclude cells that were bright or negative for CD31. Based on comparison with Quantibrite Phycoerythrin (PE) beads (BDIS), CD45RA^+^CD31^+bright^CD4^+^ cells and CD45RA^+^CD31^+dim^CD4^+^ cells have a median staining intensity of 5700 and 1500 molecules of PE, respectively [37], [38]. The purities for sorted CD4^+^ T-cells were all >99% and the subsets of CD4^+^ T-cells were >95% pure.

### Measurement of Telomere length

Genomic DNA was extracted from PBMC using the DNeasy Tissue Kit according to manufacturer's instructions (Qiagen, Valencia, California). Real-Time PCR was performed on a total of 5 ng of DNA per sample using IQ Sybr Green Supermix per manufacturer's instructions (Biorad, Hercules, California) and a previously published quantitative telomere PCR protocol [39]. The primers used were: Tel 1b: 3′-CGGTTTGTTTGGGTTTGGGTTTGGG TTTGGGTT TGGGTT-5′ and Tel 2b: 3′-GGCTTGCCTTACCCTTACCCTTACCCTT ACCCTTACCCT-5′. The beta hemoglobin primers (used to quantify cell numbers) were: HGB 1: 3′-GCTTCTGACACAACTGTGTTCACTAGC-5′ and HGB 2: 3′-CACCAACTTCATCCACGTTCACC-5′. Genomic DNA extracted from a B lymphoblastoid cell line (BLCL) and from 1301 cells, a T-cell leukemia cell line with extremely long telomere lengths (>25 kb) [40], were included in each PCR reaction to control for inter-assay variation. A no-template control was included in all PCR reactions, and data from all samples were expressed as a percentage of the telomere length of the 1301 cells, as described previously [27].

### Measurement of TREC concentrations

TREC concentration was assessed by real-time PCR protocol as described [41] and modified by us [42], [43]. Briefly, cell lysates were prepared by incubation of the cells with 100 µg of proteinase K (Boehringer Ingelheim, Petersburg, Va.) per ml for at least 1 hour at 56°C. Proteinase was inactivated by heating the sample for 10 minutes at 95°C. TREC were quantified by real-time PCR analysis, using the 5′ nuclease (TaqMan) assay and the ABI Prism 7700 sequence detector system (PE Biosystems) as described [43]. A 25-µl PCR mixture was used consisting of 5 µl of genomic DNA solution, 2.5 µl of 10x PCR buffer, 1.75 µl of 50 mM MgCl_2_, 1 µl of 5 mM deoxynucleoside triphosphates, 1 µl each of 12.5 pM forward (3′-CACATCC CTTTCAACCATGCT-5′) and reverse (3′-GCCAGCTGCAGGGTTTAGG-5′) primer and 1 µl of 5 µM probe (3′-FAM-ACACCTCTGGTTTTTGTAAAGGTGCCCACT-Tamra/QSY-5′) (MegaBases), 0.25 µl of 10 µM ROX reference dye (Invitrogen), and 0.125 µl of platinum Taq polymerase. The TREC standard consisted of known concentrations (from 20/5μl – 2×10^6^/5μl) of a plasmid containing a sjTREC fragment (kindly provided by D. Douek). Standard curves and TREC concentrations in test samples were determined using the software supplied with the ABI 7700. The number of cells in each test sample was determined by using real-time PCR to amplify β-actin DNA sequences (Applied Biosystems) per the manufacturer's instructions. All TREC and β-actin analyses were performed in triplicate on the same plate. The average variability of the triplicates was 9% for TREC measurements and 6% for the β-actin measurements. The mean value for each triplicate was used for statistical analyses.

### Statistical Analysis

The cross-sectional analysis examined the joint effects of age and HIV-1 serostatus on cell count and telomere length. Two-way ANOVAs with main effects for age and serostatus and an interaction term were followed, when significant, by post-hoc t-tests for comparisons within each of the following compartments: CD45RA^+^CD31^+^, CD45RA^+^CD31^-^, and CD45RA^-^mem (this subset contains effector/memory cells). To assess relative differences between SP and SN subjects, we computed the ratios of the average cell counts for these subgroups (SN to SP) within each age range and compartment. To make comparisons across compartments, we also computed the differences in the ratios for CD45RA^+^CD31^+^ vs. CD45RA^+^CD31^-^, CD45RA^-^mem vs. CD45RA^+^CD31^-^, and CD45RA^+^31^+^ vs. CD45RA^-^mem in each age range. Since the distribution of these ratios could not readily be determined analytically, we employed a bootstrap resampling procedure to obtain empirical p-values for the tests of cross-compartment differences [44] as follows. First we generated 20,000 replicates of the original data set, sampling subjects with replacement. For each of these “bootstrap” data sets we computed the subgroup means, the ratios of SN to SP means within each age range and compartment, and the corresponding differences in ratios across compartments. We thus obtained an empirical distribution for each of these quantities. Evidence of a significant cross-compartmental difference in SN:SP ratio is based on whether the corresponding empirical distribution lay almost entirely above (or below) 0. A 95% confidence interval for the difference was obtained by taking the 2.5% and 97.5% quantiles of the empirical distribution. Similarly, we obtained two-sided p-values for the ratio differences by doubling the tail probability corresponding to where the empirical distribution crossed 0. We took advantage of the same bootstrap simulations to validate the p-values from ANOVA using the empirical distributions of the differences in group means (analogous to t-tests). The bootstrap analyses were performed in the R programming language.

To assess differences in TREC number between subjects with high and low CD31 expression levels we used the non-parametric two-sided Wilcoxon rank-sum tests due to the small sample sizes in these subgroups. These analyses were performed using SAS, version 9.1 (Cary, NC).

To assess changes in absolute cell counts post-ART, we fit a linear mixed effects regression model. For absolute cell counts over time, the model was the same as a standard linear regression, except that a more sophisticated covariance structure was used to account for the relationships among the repeated measures within each subject. A significant positive coefficient of time would suggest that cell counts increased post-ART, while a zero or negative coefficient would suggest the opposite. Models were fit using SAS PROC MIXED with a compound symmetric covariance structure and time post-ART treated as a categorical variable.

## Results

### Preferential loss of CD31^-^ CD4^+^ naïve T-cells during HIV-1 infection

**Figure 1:**
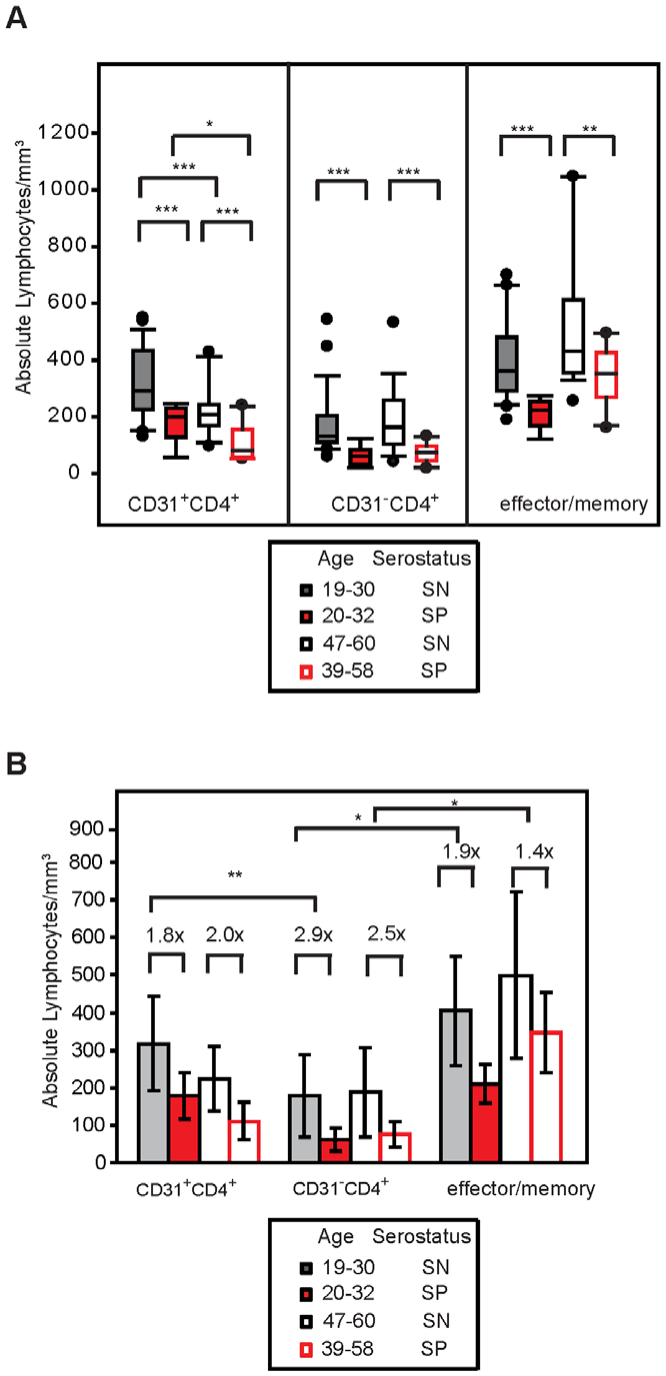
ART-naïve seropositive individuals 1-3 years post-infection have significantly fewer naïve CD4^+^ T-cells than seronegative controls. PBMC from each individual in our cross-sectional study were analyzed for the CD4^+^ naïve T-cell subsets, defined as CD45RA^+^CD27^+^ and either CD31^+^ or CD31^-^, and for the CD4^+^ T-cell effector/memory subset, defined as CD45RA^-^CD27^-^, using flow cytometry. A.) The distribution of the absolute number of naïve and effector/memory CD4^+^ T-cells was determined for each serotype and age group. B.) The average absolute number of cells of each subset was determined for each serotype and age group. For each subset, the fold difference between the seronegative and seropositive age-matched groups is shown above the bars. The asterisks signify the following values, * p<0.05, ** p<0.01, ***p<0.005.

We first quantified the percentage and absolute numbers of CD31^+^CD45RA^+^CD27^+^CD3^+^CD4^+^ (CD31^+^CD4^+^ naïve) and CD31**^-^**CD45RA^+^CD27^+^ CD3^+^CD4^+^ (CD31**^-^**CD4^+^ naïve) T-cells in the peripheral blood of young (20-32 years of age) and older (39–58 years of age) HIV-1-infected adults and age matched SN controls. As shown in Figure 1A, both older age and HIV-1 infection were associated with significantly decreased numbers of CD31^+^CD4^+^ T-cells. Young HIV-1-infected individuals demonstrated an average of 139 fewer CD31^+^CD4^+^ T-cells/mm^3^ than SN age-matched controls (317 cells/mm^3^ versus 178 cells/mm^3^, respectively; p<0.0002), and in older SP individuals, HIV-1 was associated with an additional difference of 113 cells/mm^3^ in this subset beyond what was accounted for by aging alone (223 cells/mm^3^ versus 110 cells/mm^3^, respectively; p = 0.0004). Since there was no evidence of an interaction between age and HIV-1 infection (p = 0.6423), the effect of HIV-1 infection on CD31^+^CD4^+^ T-cell numbers appears to be additive to the effects of aging alone. Notably, the absolute number of CD31^+^ CD4^+^ naïve T-cells in young SP individuals closely matched those of SN participants who were 17 to 28 years their senior (Figure 1A).

HIV-1 infection, but not age, was associated with a significant reduction of CD31^-^CD4^+^ naïve T-cells. While younger SN individuals had an average of 178 cells/mm^3^ CD31^-^CD4^+^ naïve T-cells, a decrease of 117 cells/mm^3^ was observed in the younger SP individuals (p<0.0002). However, this effect was independent of age; younger and older SP men had similar absolute cell numbers (61 cells/mm^3^ and 75 cells/mm^3^, respectively; interaction p = 0.9455). Aging in the absence of HIV-1 infection was not significantly associated with a difference in the number of CD31**^-^**CD4^+^ naïve T-cells (an average of 178 cells/mm^3^ and 188 cells/mm^3^ in the younger and older groups respectively; interaction p = 0.7476), consistent with previous reports by our group [27] and others [45], demonstrating that HIV-1 infection is associated with negative effects on this subset that are distinct from aging.

Of note, the decrease in the absolute number of CD31**^-^**CD4^+^ naïve T-cells associated with HIV-1 infection was greater than the decrease observed for any other CD4^+^ T-cell phenotype tested (Figure 1B). The number of CD31**^-^**CD4^+^ naïve T-cells was 2.9 times lower in young SP participants than in young SN participants (p = 0.0070). This is in contrast to a 1.8- and a 1.9-fold difference in the numbers of CD31^+^CD4^+^ naïve and CD45RA**^-^** effector/memory T-cell subsets, respectively, between the same two groups of participants (Figure 1B). There was also a larger difference in CD31^-^CD4^+^ naïve T-cell number between older SN and older SP participants than in the CD31^+^CD4^+^ subset for these two groups, 2.5-fold versus 2.0-fold difference respectively, but it did not reach significance. Whereas the loss of either naïve CD4^+^ T-cell subsets is likely to be detrimental to the host, our previous study has shown that the CD45RA^+^CD31**^-^** subset is particularly important for maintaining the naïve CD4^+^ T-cell pool during aging [27].

### HIV-1 infection is associated with shortened telomeres within naïve CD4^+^ T-cells

**Figure 2:**
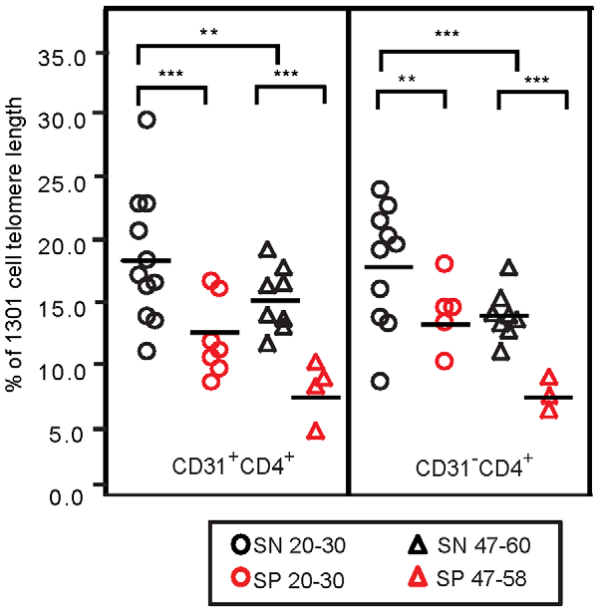
Telomeres are significantly shortened in naïve CD4^+^ T-cells within 1-3 years of HIV-1 infection. PBMC from each individual in the cross-sectional study were sorted into CD31^+^CD4^+^and CD31^-^CD4^+^ T-cell subsets. The sorted cells were then subjected to telomere length Real-Time PCR. Telomere length values are expressed as a percentage of telomere lengths within a human T-cell leukemia cell line, 1301 cells. Each symbol represents a sample from a single individual within the indicated serostatus and age group. The asterisks signify the following values, * p≤0.05, ** p≤0.01, *** p<0.005.

As shown in Figure 2, HIV-1 infection was associated with significant telomere shortening within both subsets of naïve CD4^+^ T-cells across both age groups (younger: p = 0.0004 for CD31^+^, p = 0.0096 for CD31^-^; older: p<0.0002 for both naive subsets). In agreement with our previous findings [27], aging was also associated with telomere shortening in both naïve CD4^+^ T-cell subsets, (younger vs. older SN: p = 0.0132 for CD31^+^ and p = 0.0018 for CD31**^-^** subsets; younger vs. older SP: p = 0.0036 for CD31^+^ and p<0.0002 for CD31^-^). There was no evidence of an interaction between age and HIV-1 infection in either naïve subset (interaction p = 0.9564 for CD31^+^ and p = 0.5492 for CD31**^-^** CD4^+^ naïve T-cell subsets) indicating that, although age and HIV-1 serostatus each contribute to telomere shortening, these effects appear to be additive.

### CD31 expression correlates with TREC number in CD31^+^CD4^+^ naive T-cells

**Figure 3:**
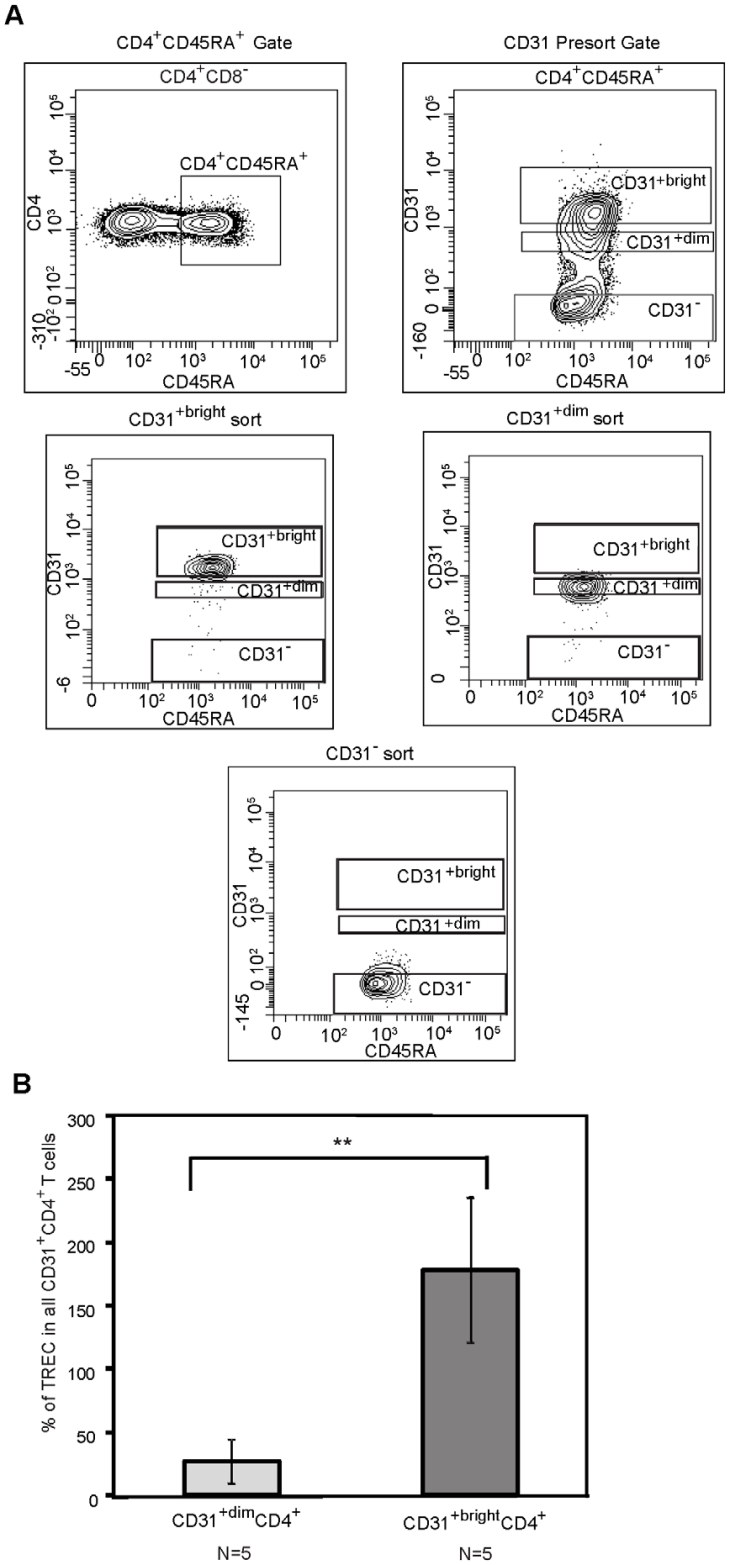
TREC content correlates with levels of CD31 expression on CD31^+^CD4^+^ naive T-cells. Fresh PBMC from five HIV-1 seronegative individuals, ranging from 25-56 years of age, were sorted into total CD31^+^CD4^+^, CD31^+bright^CD4^+^ and CD31^+dim^CD4^+^ naïve T-cell subsets. A.) Pre-sort gates for determining the CD31^+bright^CD4^+^, CD31^+dim^CD4^+^, and CD31^-^CD4^+^ naïve T-cell subsets are shown. The bottom three plots depict representative post-sort analysis of the subsets. B.) Genomic DNA was extracted from each subset and subjected to quantitative Real-Time PCR to quantify TREC content. The data is expressed as a percentage of TREC number in the indicated subset shown relative to TREC number in total CD31^+^CD4^+^ naïve T-cells. The asterisks signify the following value, ** p = 0.009.

Due to the challenge of obtaining the quantity of CD31^+^ CD4^+^ naïve T-cells from both older adults and HIV-1-infected individuals required for TREC and telomere analysis, we investigated alternative methods to determine the proliferative history of CD31^+^CD4^+^ naïve T-cells for use in the experiments described below. Based on the observation that CD31 is eventually lost from the surface of CD31^+^ naïve CD4^+^ T-cells [32], [46], we investigated whether the relative fluorescence intensity of CD31 on CD31^+^CD4^+^ naïve T-cells was associated with differing levels of TREC content. PBMC from five seronegative participants were sorted into three subsets based on the relative number of CD31 molecules on the surface of the cells: CD31^+bright^CD4^+^, CD31^+dim^CD4^+^, and total CD31^+^CD4^+^ (Figure 3A). As shown in Figure 3B, the level of CD31 expression was, in fact, strongly associated with TREC number in CD31^+^CD4^+^ naive T-cells. The CD31^+bright^CD4^+^ naïve T-cells showed an average of 80% more TREC than were found within total CD31^+^CD4^+^ naïve T-cells, while the CD31^+dim^CD4^+^ naïve T-cell subset showed an average of 60% fewer TREC (p = 0.0088). These data suggest that CD31^+bright^CD4^+^ naive T-cells, as compared to CD31^+dim^CD4^+^ naïve T-cells, are more highly enriched in RTE that have not undergone proliferation. Thus, relative fluorescence intensity can be used as an indirect measure of proliferative history within RTE in situations where cell numbers are limiting.

### Successful ART treatment does not fully reconstitute the CD31^-^CD4^+^ naïve T-cell subset

**Figure 4:**
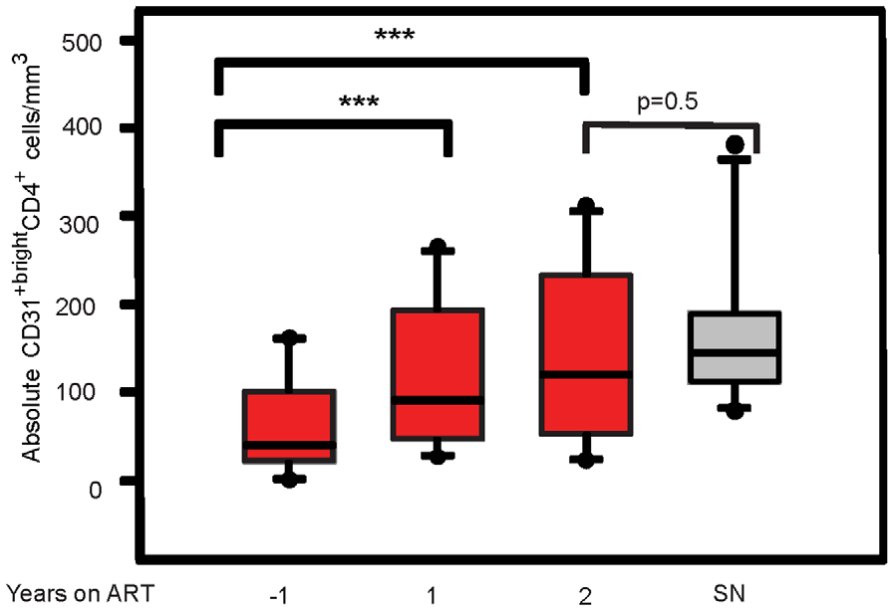
Seropositive individuals two years post-ART have significantly greater numbers of CD31^+bright^ cells. Cryopreserved PBMC from ten HIV-1 seropositive MACS participants collected at the indicated times pre- and post-ART were analyzed by flow cytometry for expression of CD4, CD45RA, and CD31. Absolute numbers of CD31^+bright^CD4^+^ naïve T-cells were determined for each participant at the indicated time points. The data is shown in a box plot format where the top and bottom of the box represent the 25^th^ and 75^th^ percentile and middle band represents the 50^th^ percentile. The asterisks signify the following value, *** p<0.005.

Since we determined that HIV-1 infection has detrimental effects on the absolute numbers of both naïve CD4^+^ T-cell subsets, we evaluated whether ART was able to restore these subsets to age-appropriate levels. Using the CD31 marker as an indicator of emigration of naïve cells from the thymus, we performed a longitudinal study on cryopreserved PBMC obtained from MACS participants before, and after, initiation of ART. One year post-ART, the absolute number of CD31^+bright^CD4^+^ naïve T-cells significantly increased from an average of 60 cells/mm^3^ to an average of 180 cells/mm^3^ (Figure 4, p = 0.0013). In fact, 2 years post-ART there was no significant difference in cell number between SP men and their SN age-matched controls (p = 0.4904).

**Figure 5:**
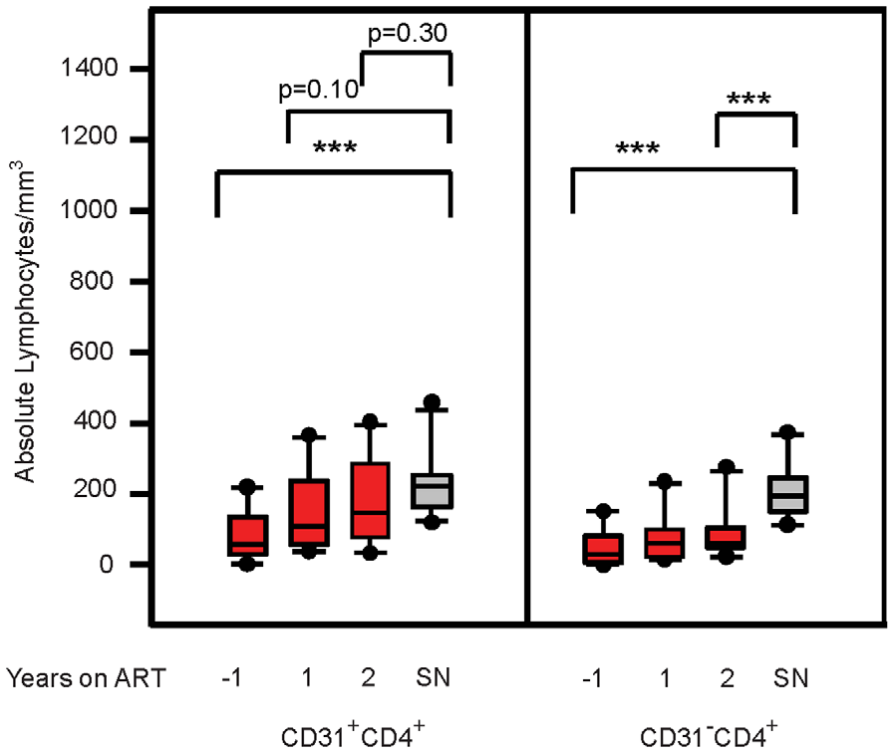
Seropositive individuals demonstrate evidence of post-ART reconstitution in CD31^+^CD4^+^, but not CD31^-^CD4^+^ T-cells. Cryopreserved PBMC from ten seropositive MACS participants collected at the indicated time points pre- and post-ART, were analyzed for the CD31^+^ and CD31^-^ naïve CD4^+^ T-cell subsets by flow cytometry. Cryopreserved PBMC from ten age-matched seronegative participants in the MACS were used as controls. The data is shown in a box plot format where the top and bottom of the box represent the 25^th^ and 75^th^ percentile and middle band represents the 50^th^ percentile. The asterisks signify the following value, *** p<0.005.

Since CD31^+bright^CD4^+^ naïve T-cells appear to be enriched for non-proliferated RTE (Figure 3B), the data in Figure 4 are consistent with previous reports of measurable thymic emigration after only one year of ART [43], [47] and continuing after that time. Interestingly, although there was no significant difference between the CD31^+^CD4^+^ naïve T-cell subset and SN controls after 2 years of ART (p = 0.2670), the absolute number of CD31**^-^**CD4^+^ naïve T-cells at the same time point showed no significant increase (Figure 5). The ultimate outcome, therefore, is a significant difference in CD31^-^CD4^+^ T-cell numbers between SP and SN age-matched controls two years post-ART (p = 0.0022).

## Discussion

Our results demonstrate that the negative effects of HIV-1 infection and aging on naïve CD4^+^ T-cells are predominantly additive. However, HIV-1 infection also exerts a distinct negative effect on CD31^-^CD4^+^ T-cell number that is not seen with aging. Together, these results suggest a role for impaired helper T-cell responses, particularly to neoantigens, in the more rapid progression to AIDS observed in individuals 50 years of age or older.

Older SN individuals demonstrate diminished absolute numbers of CD31^+^CD4^+^ naïve T-cells and shortened telomeres within those subsets [27]. Both of these deficits undoubtedly contribute to the well-documented reduced responses to vaccines and other neoantigens well documented in older adults [29]–[31]. Indeed, initial HIV-1 infection of older individuals is likely to be met with a diminished CD4^+^ T-cell response, which, in turn, would affect both B- and T-cell responses to HIV-1, allowing the virus to spread quickly. Moreover, our results suggest that once infection is established, the naïve CD4^+^ T-cell compartment is further impaired by an accelerated loss of CD31^+^CD4^+^ naïve T-cells, and a loss of CD31^-^ naïve T-cells that is not normally associated with SN adults in their fifties [27]. Compounding this cell loss is the additive effect of aging and HIV-1 infection on telomere shortening within both subsets of naïve CD4^+^ T-cells (Figure 2). Since telomere length is associated with proliferative capacity, those naïve CD4^+^ T-cells still present after the initial HIV-1 infection would be unlikely to generate, or support, robust CD8^+^ T- and B-cell responses to HIV-1 or opportunistic infections. Not only would HIV-1 be allowed to replicate more rapidly in older adults, but there would also be less CD4^+^ T-cell reserves, due to the reduced numbers of CD4^+^ T-cells and their progeny. These effects would undoubtedly contribute to the more rapid progression to AIDS.

The underlying mechanisms for the accelerated loss of the naïve CD4^+^ T-cells during HIV-1 disease most likely include increased recruitment into the effector/memory pool as well as direct infection with HIV-1 and eventual cell death. The latter is supported by previous reports showing that naïve CD4^+^ T-cells can be infected by HIV-1 [48], [49]. Our own unpublished observation that the CD31^-^ naïve subset harbors HIV-1 *in vivo* in ART-naïve individuals (data not shown) may also partially explain why this subset declines in HIV-1-infected, but not age matched SN, individuals.

Loss of the CD31^-^CD4^+^ naïve T-cells subset, and failure of this subset to reconstitute, is likely to have long-term deleterious effects on the immune response to neoantigens in individuals treated with ART. We previously demonstrated that homeostasis of the naïve CD4^+^ T-cell subset in adults is largely maintained by proliferation of CD31^-^CD4^+^ naïve T-cells and not by recent thymic emigrants (CD31^+^CD4^+^ naïve T-cells) [27]. Furthermore, the presence of clonal expansions of naïve CD31^-^CD4^+^ T-cells in HIV-1-infected individuals, similar to those in the effector/memory CD4^+^ T-cell pool, suggest that it is the CD31^-^CD4^+^ T-cells which are recruited into the effector/memory pool in response to antigen (Kilpatrick, et al. unpublished results). Failure of this subset to fully reconstitute in the older SP group within 2 years after ART initiation (Figure 5) suggests that, despite reconstitution of the CD31^+^ naïve CD4^+^ T-cell compartment shown by us and others [50], fewer naïve CD4^+^ T-cells are available for recruitment into the effector/memory pool, as compared to uninfected peers. The lack of reconstitution could be due to a true “block” in differentiation from CD31^+^CD4^+^ to CD31^-^CD4^+^, or by CD31^-^CD4^+^ T-cells being rapidly recruited into the effector/memory pool to fill the “immunologic space”. These two mechanisms would have very different implications for the health of ART-treated individuals and could suggest very different therapeutic strategies for enhancing overall CD4^+^ T-cell reconstitution.

To our knowledge, this is the first study demonstrating shortened telomere lengths in naïve CD4^+^ T-cells sorted from HIV-1-infected participants and subdivided by the surface markers CD31 and CD27. While these data accord with the majority of studies examining telomere length in total, or effector/memory, CD4^+^ T-cell populations during HIV-1 infection [51]–[54], they do conflict with the findings of Miedema and colleagues [55]. Using CD45RA and CD45RO to sort naïve and effector/memory cells, Wolthers et al. [55] failed to find evidence of telomere shortening in either CD4^+^ T-cell subset during HIV-1 infection. The difference between the Wolthers et al. results and our own study may be due, at least in part, to a difference in flow cytometry gating strategies. For example, expression of CD45RA is not a stringent phenotype for naïve CD4^+^ T-cells; other cell types, such as terminally differentiated cells would be included in this population. In addition, in contrast to the Wolthers, et al. study, our own HIV-1 infected cohort was age-matched to the SN controls to avoid the confounding effects of aging on the telomere lengths within our HIV-1 infected individuals.

Our observation of telomere shortening in even the least differentiated naïve CD4^+^ T-cells (i.e.,the CD31^+^CD4^+^ T-cell subset) in response to both aging and HIV-1 infection (Figure 2) is intriguing and implies possible telomere shortening in an earlier progenitor cell. In support of this hypothesis, both HIV-1 infection and aging have been linked to decreased telomerase activity within hematopoietic progenitors [56]–[58]. Alternatively, it is possible that extensive cellular proliferation, oxidative stress, or a deficit in telomerase directly contributes to telomere shortening within the peripheral CD4^+^ naïve T-cells. Our data do not support a major role for extensive proliferation of the naïve T-cells alone in the telomere shortening. Based on the correlation between TREC levels and CD31 expression (Figure 3B), we performed a cross-sectional analysis of CD31 expression levels on naïve CD31^+^ CD4^+^ T-cells from older vs. younger SN and HIV-1 infected age matched adults, but did not find a diminution of the CD31 relative fluorescence intensity that would be consistent with extensive cellular turnover during aging or HIV-1 infection (data not shown). In addition, in a previous cross-sectional study of the naïve CD4^+^ T-cell compartment during aging, we observed a significant decline in TREC in naïve CD31^+^ T cells over a 1-2 decade period [27]. However, the T-cell expansion was subtle and unlikely to completely account for the significant telomere shortening associated with aging, suggesting that additional factors, possibly oxidative stress, may be involved. Indeed, oxidative stress is associated with chronic inflammation, which is well documented in both HIV-1 infection [59] and aging [60]–[63] and is known to accelerate telomere shortening in both the presence and absence of proliferation [64], [65].

Our results lead us to hypothesize that the additive effects of HIV-1 and aging on the CD31^+^CD4^+^ naïve T-cell subset, and the HIV-1 associated loss of the CD31^-^ naïve CD4^+^ T-cells, contribute to accelerated HIV-1 disease progression in HIV-1-infected adults over the age of 50 [66], [67], and to the decreased response to anti-retroviral therapy in older HIV-1-infected adults [68], [69]. Moreover, there is accumulating evidence that ART-treated HIV-1-infected individuals clinically present with malignancies and infectious diseases more consistent with older SN adults [3]–[5], [70], [71]. Further elucidation of the mechanisms contributing to naïve CD4^+^ T-cell loss, telomere shortening, and delayed or arrested reconstitution of the CD31^-^ naïve CD4^+^ T-cell subset may lead to the identification of novel therapeutic targets to enhance the immune response against HIV-1 and other pathogens, as well as strategies to retard the formation of neoplasms in ART-treated individuals.
